# Efficacy of Methotrexate in patients with plaque type psoriasis

**DOI:** 10.12669/pjms.305.4451

**Published:** 2014

**Authors:** Sabiqa Haider, Zarnaz Wahid, Najam-us-Saher, Farzana Riaz

**Affiliations:** 1 Sabiqa Haider, Post-Graduate, Department of Dermatology, Dow University of Health Sciences, Karachi, Pakistan.; 2 Zarnaz Wahid, Professor & Head of the Department of Dermatology, Dow University of Health Sciences and Civil Hospital, Karachi, Pakistan.; 3 Najam-us-Saher, Specialist (Classified) Dermatologist, Shaukat Omar Memorial Hospital, Fauji Foundation, Karachi, Pakistan.; 4 Farzana Riaz, Senior Registrar, Department of Dermatology, Children Hospital, Lahore, Pakistan.

**Keywords:** Plaque psoriasis, Methotrexate, Efficacy, Remission

## Abstract

*Objective:* To assess the efficacy of Methotrexate in patients with plaque type psoriasis.

***Methods:*** This descriptive study was conducted in the department of Dermatology, Civil Hospital Karachi from September 2009 to March 2010. Seventy three patients between 18 to 50 years of age suffering from plaque type psoriasis with PASI score of >10 were included in the study after taking the informed consent. Oral methotrexate in a dose of 7.5 mg/week was given for 8 weeks. The data collected included demographic profile (age and gender), duration of disease, site of involvement, size of plaque, severity of plaque measured by Psoriasis Area and Severity Index (PASI) score before starting the treatment and at the end of treatment. Efficacy was labeled with a PASI score of ≤5 at the end of 8 weeks.

***Results: ***Out of 73 patients there were 45 (61.6%) males and 28 (38.4%) females. The mean ±SD age was 40.0±12.6 years. The mean baseline PASI score showed clear and comparable improvement from a mean ± SD PASI score of 14.8±4.2 to 4.9±4.3.Twenty nine (40%) patients had an almost complete remission during the 8 weeks of treatment. Partial remission was achieved in 44 (60%) patients. The clearance time for psoriasis ranged from 5-7 weeks (mean 6±0.89 weeks).

***Conclusion: ***Treatment with methotrexate for chronic plaque psoriasis brings satisfactory disease control and improved quality of life.

## INTRODUCTION

Psoriasis is a common, chronic, inflammatory and proliferative skin disorder in which both genetic and environmental factors have a critical role.^1^^,^^2^ The characteristic lesions consist of red, scaly, sharply demarcated, indurated plaques, present particularly over extensor surfaces and scalp.^3^^,^^4^ Chronic plaque psoriasis has an estimated worldwide prevalence of 0.1-3%.^5^ Upto 50% of patients may enter in remission phase spontaneously for different periods of time.^6^

The objective of treatment of psoriasis is to gain initial and rapid control of disease process, maintain long-term remission and improve quality of life.^2^^,^^7^ Although there is no cure for psoriasis but several treatments can minimize the disease and some can also induce remission of months to years.^8^ Topical therapy is considered as the first line therapy for psoriasis, however many patients do not respond or have extensive disease. Systemic therapy is required for these patients, which include photochemotherapy, conventional systemic, and more recently biological agents.^2^^,^^9^^,^^10^

In developing country like Pakistan, methotrexate is an economical and effective antipsoriatic agent and is given for extensive plaque type psoriasis, erythrodermic, acute pustular and severe psoriatic arthropathy.^11^It acts by inhibiting DNA synthesis by competitive inhibition of dihydrofolatereductase and thus have an anti-mitotic action on psoriatic skin.^9^ Methotrexate was originally used in psoriasis due to its effect on rapidly dividing keratinocytes, however it also has anti-inflammatory and immunomodulating properties. Methotrexate still represents a treatment option with good efficacy and tolerance especially in poor countries.^11^

Based on the available clinical data it is suggested that the methotrexate may reduce the severity of psoriasis in at least 75% of patients.^12^ Psoriasis is a common skin disorder and extensive disease generally does not respond to the topical treatment. Facilities for the phototherapy are not widely available in our country and other systemic treatment option are either expensive or have significant side effects. The rationale of the study was to find out the efficacy of methotrexate so that it could be used in subsequent patients as a cost effective treatment modality.

## METHODS

This descriptive study was conducted in the department of Dermatology, Civil Hospital Karachi from 9-09-2009 to 08-03-2010 over a period of six months. The synopsis was approved by Research Training and Monitoring Cell, College of Physicians and Surgeons Pakistan. Non-probability purposive sampling was used for sample collection. Patients between 18 to 50 years of age suffering from plaque type psoriasis of any duration with any severity of Psoriasis Area and Severity Index (PASI) score >10 were included in the study. Patients taking other treatments for the disease, suffering from anemia, thrombocytopenia, leukemia, active infection (e.g. tuberculosis, septicemia), peptic ulcer disease, renal or hepatic disease, cardiovascular disease and alcoholism were excluded. Pregnant women and lactating mothers were also excluded. Patients who were already taking treatment for psoriasis with Methotrexate or any other medicine were not included.

Patients presenting to Out Patients Department (OPD) of Dermatology, Dow University of Health Sciences & Civil Hospital, Karachi, with diagnosis of plaque type psoriasis fulfilling the inclusion criteria were included after taking informed consent. Oral methotrexate in a dose of 7.5mg per week was prescribed. The data was collected on a predesigned proforma from each patient and included demographic profile (age and gender), duration of disease, site of involvement, size of plaque, severity of plaque measured by PASI score before starting the treatment and efficacy. Baseline and weekly complete blood count (CBC), urinalysis, liver enzymes, skin examination and review of systems were carried out to monitor the side effects of methotrexate. Efficacy was labeled with a PASI score of ≤5 at the end of 8 weeks.

Data was analyzed by using SPSS version 17 for descriptive statistics. The results were given in the form of frequency and percentage for qualitative data i.e. gender, site of involvement, efficacy. Mean and standard deviation for quantitative data i.e. age, severity of Plaque type psoriasis (measured by PASI score) and size of plaque.

## RESULT

A total of eighty six patients between 18 to 50 years of age (Mean ± SD 40.0±12.6) with 45 (61.6%) males and 28 (38.4%) females suffering from plaque type psoriasis with PASI score of >10 were initially included in the study. Thirteen patients did not complete the 8-week treatment because of reversible elevations in liver enzyme levels (the highest level measured was an alanine aminotransferase level of 198 U per liter) and were excluded from the study.

Majority of the patients had skin types IV or V. The predominant symptom was itching followed by joint pains. Sunlight with summer exacerbation was provocative factor in four patients, Koebner phenomenon was seen in three patients. No other precipitating factor was detected.

The Psoriasis Area and Severity Index (PASI) was used to assess clinical status. Baseline and weekly PASI scores of all patients were maintained. The efficacy was labeled with a PASI score of < 5 at the end of 8 weeks. The mean baseline PASI score before treatment was 14.8±4.2 (Table-I). Patients were given oral methotrexate in a dose of 7.5 mg per week.

The overall rate of response was high with 68 (93%) of 73 patients had reached the threshold for a minimal response - a 25 percent reduction from base line in scores after 8 weeks of treatment, before the dose was tapered. After 8 weeks, the primary outcome measure of disease activity (PASI score) showed clear and comparable improvement from a mean (SD) PASI score of 14.8±4.2 to 4.9.0±4.3 (Table-I).

Twenty nine (40%) patients had an almost complete remission (defined as a reduction in the base-line score for the psoriasis area-and-severity index of more than 90 percent) during the 8 weeks of treatment. Partial remission (defined as a reduction in the base-line score of more than 75 percent) was achieved in 44 (60%) patients. The clearance time for psoriasis ranged from 5 - 7 weeks (mean 6±0.89 weeks).

## DISCUSSION

The present study demonstrated a good efficacy of oral methotrexate in the treatment of plaque psoriasis. Methotrexate and cyclosporine are often used in daily clinical practice, but which of the two is more effective has not been established.^5^ Although we have not compared the two drugs in this study, results of this study showed that methotrexate can be used with good results.

**Table-I:** Efficacy of oral methotrexate on the Study Subjects

***Variable***	***Mean***	***Standard Deviation (SD)***
Psoriasis Area and Severity Index (PASI)		
Baseline	14.8	4.2
At the end of 8 weeks	4.9	4.3
Clearance time for psoriasis (weeks)	6	0.89

The current management of severe psoriasis is based on the principles of rotational therapy, which stresses frequent alternations in treatment approaches in order to reduce the cumulative risk of side effects.^2^ The choice of treatment is influenced by short-term as well as long-term considerations, including the severity of the disease, the effectiveness of a given medication and its side effects, the patient's quality of life and the ease of treatment. According to the guidelines for the treatment of psoriasis, which are based on the 1997 review, UVB should be tried first, and if it proves to be ineffective, it should be followed in order by PUVA, methotrexate, acitretin, and finally, cyclosporine.^13^^,^^14^ In latest consensus guidelines for the management of plaque psoriasis^15^ published in 2012 it was proposed that methotrexate may be used as a first-line systemic drug for plaque psoriasis and compared with cyclosporine, has a more modest effect, but can be used continuously for years or decades.

In a study done by Opmeer BC et al.^16^, base line PASI score were 13.4 ± 3.6 and at the end of 16 week were 5.0± 4.5.In this study, mean baseline PASI score before treatment was 14.8±4.2 and at the end of 8 weeks of treatment was 4.9±4.3. The results are comparable with Opmeer study.

Despite the existence of guidelines^13^^,^^14^, the regimens of both methotrexate vary substantially among countries in terms of the route (oral vs. intramuscular) and the dose. We used a starting dose of 7.5 mg of methotrexate per week. We chose this dose in the absence of evidence from dose-finding and treatment-duration studies. The guidelines, however, recommend an initial dose of 2.5 to 5 mg because of the risk of myelosuppression during the first 10 days of treatment.^13^ The 8-week treatment period we used was proved to be effective in a previous study of methotrexate, in which more than 80 percent of the study population had clinical improvement.^17^

Recently, multiple randomized control trials have been published about Secukinumab, a human anti-IL-17A IgG1κ monoclonal antibody, in patients with moderate-to-severe plaque psoriasis.^9^^,^^18^ Their initial results are promising, however cost will remain a major factor for their widespread use, an important consideration in Third World countries like Pakistan.

***Limitation:*** Although, the sample size of this study is not large and it’s not a randomized trial, still the resulted showed that low dose oral methotrexate can be used effectively for plaque type psoriasis.

## CONCLUSION

Treatment with methotrexate for chronic plaque psoriasis brings satisfactory disease control, and improved quality of life. Further long term studies are needed to assess the safety of methotrexate.

## Authors Contribution:

**NS **conceived, designed and did statistical analysis & editing of manuscript.

**SH & FR **did data collection and manuscript writing.

**ZW** did review and final approval of manuscript.

**NS **takes the responsibility and is accountable for all aspects of the work in ensuring that questions related to the accuracy or integrity of any part of the work are appropriately investigated and resolved.

## Notes

***Source of funding: ***None.
